# Mitral balloon valvuloplasty during pregnancy:The long term up to 17 years obstetric outcome and childhood development

**DOI:** 10.12669/pjms.301.4305

**Published:** 2014

**Authors:** Gulraze A, Kurdi W, Niaz FA, Fawzy ME

**Affiliations:** 1 Gulraze A, MRCOG, Perinatology Section, Department of Obstetrics & Gynecology, King Faisal Specialist Hospital & Research Centre, Riyadh, Saudi Arabia.; 2 Kurdi W, MD, Perinatology Section, Department of Obstetrics & Gynecology, King Faisal Specialist Hospital & Research Centre, Riyadh, Saudi Arabia.; 3 Niaz FA, MRCP, Department of Medicine, King Khalid University Hospital, Riyadh, Saudi Arabia.; 4 Fawzy ME, FRCP, Formerly Cardiologist, Department of Medicine, King Faisal Specialist Hospital & Research Centre, Riyadh, Saudi Arabia.

**Keywords:** Childhood development, Mitral Balloon Valvuloplasty, Maternal and fetal outcome, Pregnancy

## Abstract

***Background & Objectives*** *:* We report 17 years outcome of subsequent pregnancies of women with severe Mitral Stenosis (MS) who underwent Mitral Balloon Valvuloplasty (MBV) during pregnancy and the follow up of the children born of such pregnancies.

***Methods:*** Twenty three pregnant patients suffering from severe MS (NYHA-New York Heart Association class III/IV) who underwent MBV by Inoue balloon catheter technique during second trimester were enrolled. The study was performed between January 1992 and December 2008 at King Faisal Specialist Hospital & Research Centre, Riyadh, Saudi Arabia, during which time, details about the obstetric outcome and childhood development were recorded. Mean follow up period was 10± 5.5 years (range 1-17 years).

***Results:*** MBV was successful in all patients with improvement in their NYHA class to I/II. All patients were followed until term and had uneventful course after MBV. Twenty two (95.6%) patients delivered 23 babies including a twin birth. These children exhibited normal growth and development according to their age. Nineteen patients had further pregnancies and gave birth to 38 live & healthy babies with one still birth and no unfavorable maternal outcome. Of these, 97.4% were singleton pregnancies while 2.6% were twin pregnancies. Spontaneous abortions were recorded in 21.5% and there was one still birth (2.5%) and one ectopic pregnancy (2.5%).

***Conclusion*** ***:*** Mitral Balloon Valvuloplasty is a safe and useful procedure during pregnancy, with no short or long term adverse affects on the mothers and their obstetric future. The children born of subsequent pregnancies exhibited normal physical and mental development.

## INTRODUCTION

Rheumatic MS is the commonest cardiac valvular lesion in pregnancy. MS can cause significant maternal as well as fetal mortality and morbidity. Maternal mortality is almost 1% and varies with NYHA functional class being 0.4% for women in class I and II while 6.8% in class III and IV.^1^ The rate of maternal complications is proportional to severity of MS and ranges from 26% with mild MS (mitral valve area >1.5 cms) to 67 % with severe MS (valve area <1.0 cms).^2^ Fetal mortality also varies with functional class and may be as high as 30% in class IV.^3^ The management of MS relies on medical therapy but if it fails surgical treatment is the alternative approach. With increasing expertise in minimal invasive surgery, percutaneous mitral balloon valvuloplasty (PMBV) by Inoue technique has almost replaced surgical commissurotomy. Several reports of its immediate effects on mothers and outcome of these pregnancies have shown a favorable result.^4^^-^^6^ Few studies have reported long term outcome of pregnant mothers after MBV with favorable fetal and maternal outcome.^7^^-^^10^ We report a long term obstetrical outcome of such women and the effects of MBV on the development of children born of subsequent pregnancies.

## METHODS

This was a retrospective case series of 23 pregnant patients with MS referred to King Faisal Specialist Hospital & Research Centre, Riyadh, Saudi Arabia between January 1992 to December 2008. Their mean age was 30.93±8.15 years (range 17-42 years) and mean gestational age was 28±8 weeks (20-34 weeks). All patients were in the second trimester of their pregnancy and were symptomatic with NYHA class III/IV with mitral valve area (MVA) <1.1cm. Doppler ECHO was performed before and immediately after MBV. They underwent MBV by Inoue balloon catheter technique during the second trimester. Abdomen & pelvic shielding was maintained during the procedure to minimize radiation exposure to the fetuses. Fluoroscopy was used only during trans-septal puncture and balloon inflation. Fetal monitoring was carried out during the procedure by cardiotocography. The immediate success of MBV was assessed by increase in mitral valve area and improvement in functional NYHA class. All patients were followed until delivery during index pregnancies. Out of the total, long term data were available for 19 patients who completed the study. We report here follow up of upto 17 years (from 1992 through 2008) of the mothers who underwent MBV during pregnancy and babies born of these pregnancies. This study also includes the subsequent obstetric career of such women and future development of their off springs.

The study was approved by the Research Ethics Committee of the hospital. Data were collected by review of the medical records, telephonic and direct communication with the patients. Clinical and echocardiographic assessments of the patients were performed initially after 6 months of MBV and annually thereafter. The long term outcome was analyzed for subsequent obstetric course of the mothers and the childhood development of the babies born. Subsequent obstetric future of these women was analyzed for incidence of abortions, still births, pre-term deliveries, and congenital anomalies. Mode of delivery, birth weights and neonatal morbidity or mortality were also recorded. Chi-square test was utilized to determine any significant difference between various modes of delivery, and fetal outcome. Follow up of children was completed by verbal communication with their mothers and family by obtaining history of milestones, growth, speech, development, recurrent illnesses and any hospital admission. Their learning abilities were assessed by performances at school and compared with their peers and siblings. 

## RESULTS

***Immediate outcome:*** MBV was successful in all patients with significant improvement in their NYHA class to I/II. All 23 patients who were followed up until term had uneventful course after MBV. Among them 22 (95.6%) patients had spontaneous vaginal delivery (SVD) and one (4.3%) had Lower uterine segment caesarian delivery for fetal distress which resulted in a fresh stillbirth. One pregnancy resulted in twins. The mean birth weight was 3.02± 0.38 kg (2.5- 3.6kg). The average apgar score was 7&2 at 10 minutes. None of the babies required intensive care monitoring and no malformations were found. One baby died because of sudden infant death syndrome in the neonatal period and another died at 8 months of age due to pneumonia.^11^

***Long term maternal outcome:*** Re-stenosis of the mitral valve developed in 3 patients (16%) who had unfavorable mitral valve morphology. One of them had a repeat MBV after five years of initial MBV and other two patients underwent MV replacement 1.5 years after delivery. The remaining patients remained stable and maintained their MVA and NYHA class I/II.^11^

***Long term obstetric outcome:*** Nineteen patients had further pregnancies but one patient was put on oral contraceptive pills after an abortion. Out of the remaining, eight became pregnant at least once after the procedure and 5 got pregnant twice. Three women had 3 pregnancies and another 3 women had 4 subsequent pregnancies. Thirty nine (39) babies including one still birth were born of such pregnancies. The mode of delivery in these subsequent pregnancies was SVD in 74.4%, lower uterine segment caesarean delivery in 20.5% and vacuum extraction in 5.1% (Table-I). The difference between various modes of delivery was statistically significant (p<0.05). Most (97.4%) of these were singleton pregnancies while 2.6% were twin pregnancies. There was no maternal mortality. Majority (84.6%) ended up having healthy and alive babies and 12.8% were pre-terms (<37 weeks of gestation), while one (2.5%) still birth (>24 weeks of gestation) and one ectopic pregnancy was recorded (Table-II). The difference between various fetal outcome was statistically significant (p<0.05). Among all the conceptions, spontaneous abortions (<24 weeks of gestation) were recorded in 21.5% (11/51) including one abortion in 7 patients and two women had 2 abortions in between successful pregnancies. Among the neonates, 92.3% were of normal birth weight (2.8±0.6kg) while 7.7% had low birth weights (<2.5kg). No congenital malformations or neonatal mortality was recorded. One child died at the age of one year after gastroenteritis and another died at the age of two years due to pneumonia. The remaining thirty six children born of these women are currently alive and healthy and developing normally according to their age. None of them required any special assistance or care at home or school.

## DISCUSSION

When the medical management of MS fails to relieve symptoms, then surgical options are considered which includes MVR, open or closed M.V. commissurotomy or MBV. Over the years, management of MS has progressed from medical to open surgical therapy to minimal invasive cardiac procedure.

**Table-I:** Distribution of sample by mode of delivery (n=39).

*Mode of delivery*	*Percentage*
SVD	74.4%
Asissted Delivery (forceps/vacuum)	5.1%
LSCS	20.5%

**Table-II:** Distribution of sample by fetal outcome (n=39).

*Fetal outcome*	*Percentage*
Pre term	12.8%
Still birth	2.5%
Full term live birth	84.6%

PMBV with Inoue balloon catheter technique has superseded other surgical treatments for severe MS in pregnancy due to its simplicity and is associated with reduced cost and morbidity, short hospital stay and avoids thoracotomy and general anesthesia.^8^^,^^12^^,^^13^ Ideally, MBV should be performed prior to pregnancy, otherwise it is recommended to be performed in the second trimester to avoid radiation exposure during the first trimester. Radiation received during MBV is 0.2 rads which is far below the recommended safe dose of 5 rads.^14^During the procedure, radiation can be minimized by abdomino- pelvic shielding and by reducing the duration of fluoroscopy.

MBV for relief of symptoms due to severe MS during pregnancy has been shown to be an excellent procedure in majority of studies performed to date with over 95% success rates^7^^,^^8^^,^^9^^, ^and in some instances reaching up to 100%.^10^^,^^11^^,^^15^ The incidence of associated major complications has been considerably low ranging from 0%^11^ to 9%^16^ and continues to decline as expertise in the procedure improves with time. In this study, performance of MBV during pregnancy was not associated with any unfavorable outcome with regards to either short or long term maternal health and development of babies born from such pregnancies. There are occasional reports of MBV related perinatal mortality and preterm labour but the incidence is no different from that observed in an otherwise normal pregnant population^10^^,^^15^ One still birth due to fetal distress seen in the index pregnancy was remote from the MBV procedure. These data indicate that MBV is safe with immediate symptomatic improvement and almost no adverse effect on the outcome of pregnancy. Similar observations were made in the present study as none of the patients was adversely affected by complications associated with the procedure and most of the women had uneventful pregnancies until term. 

A number of studies have reported long term follow up of mothers ranging from 5 to 12 years^7^^,^^9^^-^^11^^,^^15^ while the duration of follow up in the present study is 17 years. Fawzy et al^11^ reported that all except three patients maintained improvement in symptoms throughout long term follow up. The 3 exceptions had unfavourable MV morphology, one of them underwent repeat MBV while in other two patients, MVR was performed.

As regards fetal outcome in subsequent pregnancies, the majority (84.6%) had full term live births, while 12.8% pre-term births in our case series are comparable to the figures of 12-13% in the USA and 5-9% in many other developed countries.^17^ The incidence of spontaneous abortions among normal women is reported to be 16.2%.^18^ In addition, the overall risk of spontaneous abortion has been reported to be 10.9% and increases with maternal age ranging from 8.7% by the age of 22 years to 84.1% by the age of 48 years or more.^19^ Over twenty one percent (21.5%) patients in the present study had spontaneous abortions which appear to be slightly higher than the reported figures. This may possibly be due to the smaller number of patients in the present study. The higher percentage of spontaneous abortions observed in the present study could also be due to relatively higher mean age of the women and is possibly not related to patients having undergone MBV. Stillbirth affects 1 in 160 pregnancies in the United States.^20^ The overall risk of stillbirth reported in the literature is 4.3 per thousand women in otherwise healthy women and that of ectopic pregnancy is 2.3%.^19^ One stillbirth (2.5%) which was due to obstetric reason and not related to MBV and only one ectopic pregnancy (2.5%) was recorded in our patients. Furthermore, the rate of twin pregnancies in the United States has been reported to be 32 per 1000 births in 2006^21^ while in our case series, there were 2.6% twin pregnancies which ended in term babies. Because of the relatively smaller number of patients in the present study it is difficult to perform a comparative analysis.

Due to different obstetrical practices, the rate of operative deliveries varies among countries and the different population groups. In a French survey for teaching hospitals, the rate of caesarean section varied from 9 to 29.5% with a national mean rate of 20.7%.^22^ The caesarean birth rate in the United States have been reported to be rising over the years from 20.7% in 1996 to 31.1% in 2006.^23^ In a study conducted in Saudi Arabia during 2006, the overall caesarean section delivery rate has been reported to be 19.1%^24^ which is comparable to the caesarean birth rate (20.5%) observed in the present study. The low birth weight in our study is 7.7% which is almost similar to 8.3% reported in USA in 2006.^25^

Childhood development was monitored up to 17 years of age in the present study which is probably the longest duration ever observed. Previous studies monitoring the development of children born of mothers undergoing MBV during pregnancy were of 3-9 years duration.^8^^-^^11^^,^^16^ The outcome of present study however was no different from the previous studies where the development of the babies remained normal. The performance of these children at school is comparable to their age-matched peers and siblings. None of them suffered from long lasting disease which needed special care or frequent hospitalizations. They did not show any evidence of radiation- induced disease or any hematological disorder.

Comparative analysis of the data collected in the present study was limited by the small number of the patients. Similarly, data describing pregnant women with MS undergoing MBV are lacking, hence comparison of our data with similar studies is not possible. It is, however, worth mentioning that the cardiac status of the patients in the present study did not deteriorate in any of the subsequent pregnancies. This indicates that MBV during pregnancy is a safe practice and is not associated with any adverse obstetric outcome.

## Author contributions:

Drs. Gulraze and Kurdi were involved in the design of the study, data collection and analysis and manuscript preparation. Dr. Niaz was involved in the data analysis and manuscript preparation. Dr. Fawzy performed the procedures and revised the manuscript.

## Notes

***Limitations of the Study: ***This study was limited by a relatively small number of patients and the conclusions drawn in the present study may not be applicable to the general population. Similarly collection of follow up data of the children through verbal communication with the mothers may have varied compared to the assessments made by the treating physicians.

## Notes

***Disclosure:*** Authors have no conflict of interest and the work was not supported or funded by any drug company.

## References

[1] Perloff JK, Pregnancy, Brauwald E (1997). cardiovascular disease. Heart Disease. A textbook of Cardivascular Medicine.

[2] Barbosa PJ, Lopes AA, Feitosa GS, Almeida RV, Silva RM, Brito JC (2000). Prognostic factors of rheumatic mitral stenosis during pregnancy and puerperium. Arq Bras Cardiol.

[3] Safian RD, Berman AD, Sachs B, Diver DJ, Come PC, Baim DS (1988). Percutaneous balloon mitral valvuloplasty in a pregnant woman with metal stenosis. Catherter Cardiovasc Diagn.

[4] Farhat MB, Maatouk F, Batbout F (1992). Percutaneous balloon mitral valvuloplasty in eight pregnant women with severe mitral stenosis. Eur Heart J.

[5] Patell JJ, Mitha AS, Hassen S (1993). Percutaneous balloon mitral valvotomy in pregnant patients with tight pliable mitral stenosis. Am Heart J.

[6] Mirsha S, Narang R, Sharma M (2001). Percutaneous transseptal mitral commissurotomy in pregnant women with critical mitral mitral stenosis. Indian Heart J.

[7] Nercolini DC, Bueno RRL, Guerios E, Tarastchuck JC, Kubrusly LF (2002). Percutaneous mitral balloon valvuloplasty in pregnant women with mitral stenosis. Cathet Cardiovasc Intervent.

[8] Routray SN, Mishra TK, Swain S, Patnaik UK, Behera M (2004). Balloon mitral valvuloplasty during pregnancy. Int J Gynaecol Obstet.

[9] Sivadasanpillai H, Srinivasan A, Sivasubramoniam S, Mahadevan KK, Kumar A, Titus T (2005). Long-term outcome of patients undergoing balloon mitral valvotomy in pregnancy. Am J Cardiol.

[10] Mangione JA, Lourenço RM, dos Santos ES, Shigueyuki A, Mauro MF, Cristovão SA (2000). Long-term follow-up of pregnant women after percutaneous mitral valvuloplasty. Catheter Cardiovasc Interv.

[11] Fawzy ME, Abdulhalim JK, Stefadouros M, Hegazy H, Kattan H, Chaudary A (2001). Long term outcome of Mitral Balloon Valvotomy in Pregnant women. J Heart Valve Dis.

[12] Iung BL, Garbarz E, Michaud P (1999). Late results of percutaneous mitral commissurotomy in a series of 1024 patients: Analysis of late clinical deterioration: Frequency, anatomic findings and predictive factors. Circulation.

[13] Fawzy ME (2009). Long-term results up to 19 years of mitral balloon valvuloplasty. Asian Cardiovasc Thorac Ann.

[14] Brent RL (1989). The effect of embryonic and factor exposure to X-ray, microwaves and ultrasound: Counseling the pregnant and non pregnant patients about these risks. Semin Onedl.

[15] Esteves CA, Munoz JS, Braga S, Andrade J, Meneghelo Z, Gomes N (2006). Immediate and long-term follow-up of percutaneous balloon mitral valvuloplasty in pregnant patients with rheumatic mitral stenosis. Am J Cardiol.

[16] Ben Farhat M, Gamra H, Betbout F, Maatouk F, Jarrar M, Addad F (1997). Percutaneous balloon mitral commissurotomy during pregnancy. Heart.

[17] Goldenberg RL, Culhane JF, Iams JD, Romero R (2008). Epidemiology and causes of preterm birth. Lancet.

[18] Mills JL, Simpson JL, Driscoll SG, Jovanovic-Peterson L, Van Allen M, Aarons JH (1988). Incidence of spontaneous abortion among normal women and insulin-dependent diabetic women whose pregnancies were identified within 21 days of conception. Engl J Med.

[19] Andersen AN, Wohlfahrt J, Christens P, Olsen P, Melbye M (2000). Maternal age and fetal loss: population based register linkage study. BMJ.

[20] Stillbirth Collaborative Research Network Writing Group (2011). Causes of death among stillbirths. JAMA.

[21] Chauhan SP, Scardo JA, Hayes E, Abuhamad AZ, Berghella V (2010). Twins: prevalence, problems, and preterm births. Am J Obstet Gynecol.

[22] Mangin M, Ramanah R, Aouar Z, Courtois L, Collin A, Cossa S (2010). Operative delivery data in France for 2007: results of a national survey within teaching hospitals. J Gynecol Obstet Biol Reprod (Paris).

[23] MacDorman MF, Menacker F, Declercq E (2008). Cesarean birth in the United States: epidemiology, trends, and outcomes. Clin Perinatol.

[24] Ba'aqeel HS (2009). Cesarean delivery rates in Saudi Arabia: A ten year review. Ann Saudi Med.

[25] Martin JA, Hamilton BE, Sutton PD, Ventura SJ, Menacker F, Kirmeyer S (2009). Births: Final data for 2006. Hyattsville, MD: National Center for Health Statistics.

